# Trust-wide improvement and standardisation of the medical handover

**DOI:** 10.1192/bjo.2021.613

**Published:** 2021-06-18

**Authors:** Kabir Yisa, Roshelle Ramkisson, Anoop Mohan

**Affiliations:** Pennine Care Foundation Trust

## Abstract

**Aims:**

Primary: To improve the quality of medical handover by increasing trust-wide completion rate for agreed quality standards from baseline to 80% by July 2019

To standardise the medical handover across all 5 boroughs within the trust by July 2019

Secondary: To separately assess the completion rate for new patient checks in relation to baseline

**Background:**

Not only is there a recognised variation in the medical handover across Pennine care foundation trust's (PCFT) 5 boroughs (Tameside, Rochdale, Bury, Oldham and Stockport), but there also appears to be diminished adherence to quality standards to a varying extent. This was shown to result from a combination of human factors and process errors consequently leading to near-miss clinical incidents. This project was therefore designed to highlight and address these issues in order to promote patient safety.

**Method:**

Having identified the problems and agreed on quality standards, baseline data were gathered through May 2019 with the primary outcome measure being the percentage completion rate for quality standards across all handovers. Changes were made to the handover document in June 2019 followed by introduction of a new handover document and post-intervention data collection through July 2019. Doctors’ satisfaction with the new handover document using a typical 5-level Likert scale was measured using a trust-wide online survey.

**Result:**

Our results showed a considerable Trust-wide improvement in the completion rate for agreed quality standards from 54% at pre-intervention to 77% at post-intervention with all 5 boroughs demonstrating improvement to varying extents. This is noted to be reasonably close to the project aim of improving the completion rate from baseline to 80%. With regards to new patient checks, there was a marginal Trust-wide increase in completion rate from 53% at pre-intervention to 56% at post-intervention. Additionally, the trust-wide online survey measuring doctors’ satisfaction with the new handover revealed a majority (82%), either strongly agreed (27.3%), or agreed (54.6%), that the new handover document has improved the overall quality of handovers.

**Conclusion:**

Trust-wide standardisation of the handover has now presented the opportunity to benchmark boroughs within PCFT against one another in terms of adherence to quality standards and alignment with best practice relating to patient safety. While substantial improvement may have been made in the overall quality of handovers across the trust, the inter-borough disparity in the extent of improvement identified gaps for future follow-up or related projects.

